# Supplementary material for: Design and implementation of digital literacy training programme: Findings of a quasi- experimental study from rural India
Source: PLOS Digit Health. 2025 Apr 10;4(4):e0000617. doi: 10.1371/journal.pdig.0000617 (PMC11984724; doi:10.1371/journal.pdig.0000617)
Supplement: Attachment — Submitted filename: renamed_b0d14.docx [file pdig.0000617.s001.docx]

#### General Comments

In this manuscript, the authors  investigates the design and effects of a digital literacy training program implemented in rural India.The study utilized a quasi-experimental research design covering assessments at multiple time points (baseline, midline, and endline) to compare differences in digital literacy levels between the intervention and control arms.Overall, it is an meaningful study.However，

MINOR revision has to be done before this manuscript could be accepted for publication in the PLOS Digital Health.

#### Major Comments

1.The introduction section provides enough background information and also cites a lot of literature to support this study, however, the statement of the innovativeness and objectives of the study is not clear enough and I would suggest a slight modification in this regard.

2. The quasi-experimental research design used in the article may lead to differences in baseline characteristics between groups due to the lack of random assignment, so it was necessary for the authors to explain how to minimize the effects of confounding factors during the data collection process.

3.Large variations in sample size across the phases, especially at the endline assessment when the sample size of the intervention group increases while the sample size of the control group decreases, may affect the comparability of the data between the two groups, and there should be a need for more detail on sample substitution to describe the criteria for selection of the substitution sample and to demonstrate that the substitution sample is similar to the original sample in terms of its baseline characteristics, thus making the sample more representative.

4.Only univariate analyses were used in the data analysis, and multivariate analyses were not fully utilized to control for confounding factors, thus failing to reveal in greater depth the impact of the interventions on bridging the digital divide.

5.Increase the discussion of the study's limitations, especially regarding the sample's limitations and the lack of long-term impact assessment.

#### Minor Comments

6.The results section of the article is sometimes cited in the wrong table, e.g., on page 16 on the comparison of socio-demographic and socio-economic characteristics of the participants should be cited as Table 1 instead of Table 2. Please check that all the data descriptions match the tables.

7.On page 18, line 13, the median score for the practical aspects of the DL communication element at the midline for the intervention and control arms was 2, not 1.Please revise it.
