# Supplementary material for: Design and implementation of digital literacy training programme: Findings of a quasi- experimental study from rural India
Source: PLOS Digit Health. 2025 Apr 10;4(4):e0000617. doi: 10.1371/journal.pdig.0000617 (PMC11984724; doi:10.1371/journal.pdig.0000617)
Supplement: Attachment — Submitted filename: Response letter.docx [file pdig.0000617.s003.docx]

**Response Letter**

**Reviewer #1: General Comments**
In this manuscript, the authors investigate the design and effects of a digital literacy training program implemented in rural India. The study utilized a quasi-experimental research design covering assessments at multiple time points (baseline, midline, and endline) to compare differences in digital literacy levels between the intervention and control arms. Overall, it is a meaningful study. However, MINOR revision has to be done before this manuscript could be accepted for publication in the PLOS Digital Health.

Response: Thanks for reviewing this manuscript, and for providing insightful feedback for its improvement.

2. The introduction section provides enough background information and also cites a lot of literature to support this study, however, the statement of the innovativeness and objectives of the study is not clear enough and I would suggest a slight modification in this regard.

Response: Page 5, Lines 117-130

3. The quasi-experimental research design used in the article may lead to differences in baseline characteristics between groups due to the lack of random assignment, so it was necessary for the authors to explain how to minimize the effects of confounding factors during the data collection process.

Response: It was difficult for us to assign the intervention randomly in the chosen area of District Gumla in Jharkhand- most of the population is tribal and represent marginalized caste groups. In the initial phase of the project, it was extremely difficult to convince the families to send their school dropout girls to be a part of this DLTP. Hence, there was a drop out and over the project period the girls got married off also. So, we tried our best in the midline and endline to keep the background characteristics similar in the intervention and control arm. We have included this as our study limitation in the discussion section.

Also, when we started this project, a digital literacy program was already in place by Google. But it was mostly for school-going children (both boys and girls). When we started the project, some of the school going girls became school dropouts and got enrolled in our program. So, it was practically very difficult to assess whether the improvement in outcomes was totally attributable to our program or was a combination of multiple projects operational in that area!! This was one reason why we didn’t attempt to do multivariate analysis- as multiple unseen confounding factors were there. So, we have tried to explain the confounders in discussion.

Line Nos- Line Nos- 444-68, Pages 19-20

4. Large variations in sample size across the phases, especially at the endline assessment when the sample size of the intervention group increases while the sample size of the control group decreases, may affect the comparability of the data between the two groups, and there should be a need for more detail on sample substitution to describe the criteria for selection of the substitution sample and to demonstrate that the substitution sample is similar to the original sample in terms of its baseline characteristics, thus making the sample more representative.

Response: Efforts were made to enroll the girls with similar background characteristics across the three phases in the intervention and control arm. But due to the type of intervention, and its implementation at community level, where the acceptance for DL was low, it led to sample heterogeneity. We have explained this as a limitation of our study in discussion section.

Line Nos- Line Nos- 444-68, Pages 19-20

5. Only univariate analyses were used in the data analysis, and multivariate analyses were not fully utilized to control for confounding factors, thus failing to reveal in greater depth the impact of the interventions on bridging the digital divide.

Response: As explained above in response No. 3.

6. Increase the discussion of the study's limitations, especially regarding the sample's limitations and the lack of long-term impact assessment.

Response: Line Nos- 444-68, Pages 19-20

7. The results section of the article is sometimes cited in the wrong table, e.g., on page 16 on the comparison of socio-demographic and socio-economic characteristics of the participants should be cited as Table 1 instead of Table 2. Please check that all the data descriptions match the tables.

Response: We have corrected this.

8. On page 18, line 13, the median score for the practical aspects of the DL communication element at the midline for the intervention and control arms was 2, not 1. Please revise it.

Response: We have corrected this

Reviewer 2

1. Dear Authors,
   Despite the crucial research on the relevant subject, in a first step, please revise the presentation of your work, particularly - in accordance with the publication standards - in "intelligible fashion and written in standard English."
   The text leaves so much unclear, and is not appropriate for a scientific review and audience. Just some examples: Title should be: "implementation of a digital..." / or programmes; rural India, not Rural India

Response: Thanks for this opportunity to revise the manuscript. We have tried our best to include the details mentioned by you.

2. Abstract: Who are "the girls" - where do they come from? What is "The project", skills about?, impact for the adolescent girls
   Proportion above median?? 50%?? Unclear, what is meant actually.
   p<0.000 does not exist.
   Overall, please go through a proofreading revision.

Response: We have included in the abstract that the girls are school droupouts from 8 blockes of Gumla district in Jharkhand,India.

Median represents the median score for various components of digital literacy indicating proportion of girls having score of DL above median value. We have incorporated these details in the methodology section.(Line no. 267-270- Page 11) Thanks.
